# Cardiac involvement in a patient with B-cell lymphoblastic lymphoma/acute lymphoblastic leukemia and a history of allogeneic hematopoietic stem cell transplantation and CAR T-cell therapy: A case report

**DOI:** 10.3389/fimmu.2022.1052336

**Published:** 2023-01-05

**Authors:** Yigeng Cao, Yadan Liu, Rongli Zhang, Weihua Zhai, Qiaoling Ma, Jialin Wei, Donglin Yang, Aiming Pang, Yi He, Xin Chen, Erlie Jiang, Sizhou Feng, Mingzhe Han

**Affiliations:** ^1^ State Key Laboratory of Experimental Hematology, National Clinical Research Center for Blood Diseases, Haihe Laboratory of Cell Ecosystem, Institute of Hematology & Blood Diseases Hospital, Chinese Academy of Medical Sciences & Peking Union Medical College, Tianjin, China; ^2^ Hematology Department of Ningbo First Hospital, Ningbo Clinical Research Center for Hematologic Malignancies, Ningbo, China

**Keywords:** B cell acute lymphoblastic leukemia, B cell lymphoblastic lymphoma, allogeneic hematopoietic stem cell transplantation, chimeric antigen receptor T cells, cardiac involvement

## Abstract

Cardiac involvement in hematological malignancies is uncommon, with only a few cases reported to date, and it often leads to a poor prognosis. Here, we report a case of a 42-year-old woman with a history of allogeneic hematopoietic stem cell transplantation (allo-HSCT) and anti-CD19 chimeric antigen receptor (CAR) T-cell therapy for B-cell lymphoblastic lymphoma/acute lymphoblastic leukemia in whom cardiac mass and myocardial infiltration were detected. Prior to this presentation, massive pericardial effusion had occurred 6 months after CAR T-cell therapy, which was improved *via* ultrasound-guided pericardiocentesis. We observed elevated cytokine levels and increased copy number of CAR DNA in both pericardial effusion and serum. Upon detecting cardiac mass and myocardial infiltration, the patient was administered tocilizumab (a humanized monoclonal antibody against IL-6 receptor), which controlled the serum cytokine levels, and reduced intensity chemotherapy, including vindesine, cyclophosphamide, and prednisolone. However, the patient finally died of multiple organ failure. To the best of our knowledge, this is the first report on the development of a cardiac mass and occurrence of myocardial infiltration after allo-HSCT and CAR T-cell therapy. This report may provide supporting data for the early diagnosis and immediate treatment of patients with cardiac involvement.

## Introduction

B-cell acute lymphoblastic leukemia (B-ALL) and B-cell lymphoblastic lymphoma (B-LBL) are precursor lymphoid neoplasms of the B lineage, which are considered to be the same disease with different developmental stages and clinical manifestations (1). According to version 1.2016 of the NCCN, less than 20% bone marrow involvement can be used as a diagnostic criterion to distinguish LBL from ALL. B-LBL is a highly malignant non-Hodgkin’s lymphoma that commonly occurs in children and adolescents. The most common occurrence sites of B-LBL are the skin, soft tissue, bone, and lymph nodes, while mediastinal or pleural involvement is rare and cardiac involvement is even rarer (2). Some studies have shown that cardiac involvement is common in secondary and malignant tumors through hematogenous metastasis, lymphatic metastasis, transvenous extension, and direct infiltration of tissues surrounding the neoplasms and that lymphoma and leukemia are the most common hematological malignancies involving the heart (3–5). The heart is hidden in the mediastinum, and there are no specific clinical manifestations of cardiac involvement, which can easily lead to misdiagnosis or missed diagnosis relatively easy in patients with cardiac metastasis.

Allogeneic hematopoietic stem cell transplantation (allo-HSCT) is currently the only available methodology to treat B-LBL, and it has been shown to greatly improve the long-term survival of patients (6). However, it is difficult to obtain a complete response (CR) or partial response (PR) in patients with B-ALL who show relapse after transplantation, and these patients exhibit an extremely poor prognosis (7). Chimeric antigen receptor (CAR) T-cell therapy is an emerging cellular immunotherapy and has revolutionized treatment modalities for relapsed or refractory B-ALL (R/R B-ALL) with a CR rate of 70%–90% (8, 9). It has thus significantly prolonged the disease-free survival of such patients (10, 11). Here, we present a case of a 42-year-old woman with an initial diagnosis of B-LBL who developed cardiac involvement after allo-HSCT and anti-CD19 CAR T-cell therapy. To the best of our knowledge, no systematic studies on the efficacy and safety of various treatments for cardiac involvement exist. This report provides relevant data for identifying cardiac involvement and selecting an appropriate treatment.

## Case presentation

A 42-year-old woman with a history of B-LBL was admitted to the Institute of Hematology and Blood Diseases Hospital, Tianjin, China, because of persistent dyspnea. In May 2019, the patient was evaluated at another hospital for recurrent pain in her right hip, which had begun 6 months earlier and gradually worsened with no obvious predisposing causes. PET-CT scans revealed multiple high-density nodules in both the kidneys, left acetabulum, left ilium, bilateral tibia, and right fibula as well as intense FDG uptake by these nodules. Immunohistochemical (IHC) analysis of a left kidney biopsy specimen indicated positivity for CD10, TdT, CD79a, Pax-5, CD43, c-myc (20%), and Ki-67 (90%) and negativity for CD3, CD20, CD5, CD23, Bcl-2, Bcl-6, MUM1, and CyclinD1. Neither bone marrow smear nor flow cytometry revealed any abnormal lymphocytes, and bone marrow biopsy revealed active proliferation of the hematopoietic tissue. Cytogenetic analysis revealed an abnormal karyotype: 46,XX,t(1,19)(q23;p13),-4,+mar[18]/46,XX(2). Gene fusion analysis showed positivity for E2A/PBX1. These findings led to the diagnosis of B-LBL with E2A/PBX1.

The patient had received two cycles of induction chemotherapy with a combination of cyclophosphamide, vincristine, doxorubicin, dexamethasone (hyper-CVAD A), methotrexate, and cytarabine (hyper-CVAD B). Bone marrow examination revealed 10% blasts, while PET-CT revealed no intense FDG uptake. Subsequently, the patient received vindesine, daunorubicin, cyclophosphamide, pegaspargase, prednisone (VDCLP), cyclophosphamide, cytarabine, 6-mercaptopurine (CAM), vindesine, cyclophosphamide, idarubicin, dexamethasone (VICD). Every bone marrow smear after each chemotherapy showed CR, while E2A/PBX1 was undetectable, 3.68% and 0.22% as determined *via* polymerase chain reaction, respectively.

The patient remained well for 3 months. In May 2020, bone marrow smear revealed 47% lymphoblasts, while the rate of E2A/PBX1 rearrangement was 45.55%. Lymphoblasts accounted for 4.96% of the marrow cellularity, as determined using flow cytometry. These results suggested a diagnosis of B-ALL with E2A/PBX1 expression. Treatment with orally administered 6-mercaptopurine and prednisone was initiated to relieve the tumor burden.

In June 2020, the patient underwent allo-HSCT from a matched, sibling donor after a conditioning regimen with cyclophosphamide, fludarabine, idarubicin, and antithymocyte globulin and total body irradiation. Early post-transplantation complications included mild diarrhea and mucositis. The bone marrow smear indicated CR, no E2A/PBX1 amplification, and full donor chimerism. PET-CT revealed that the treatment was effective and that the CR of the disease was achieved, with a Deauville score of 1 point.

**Figure 1:**
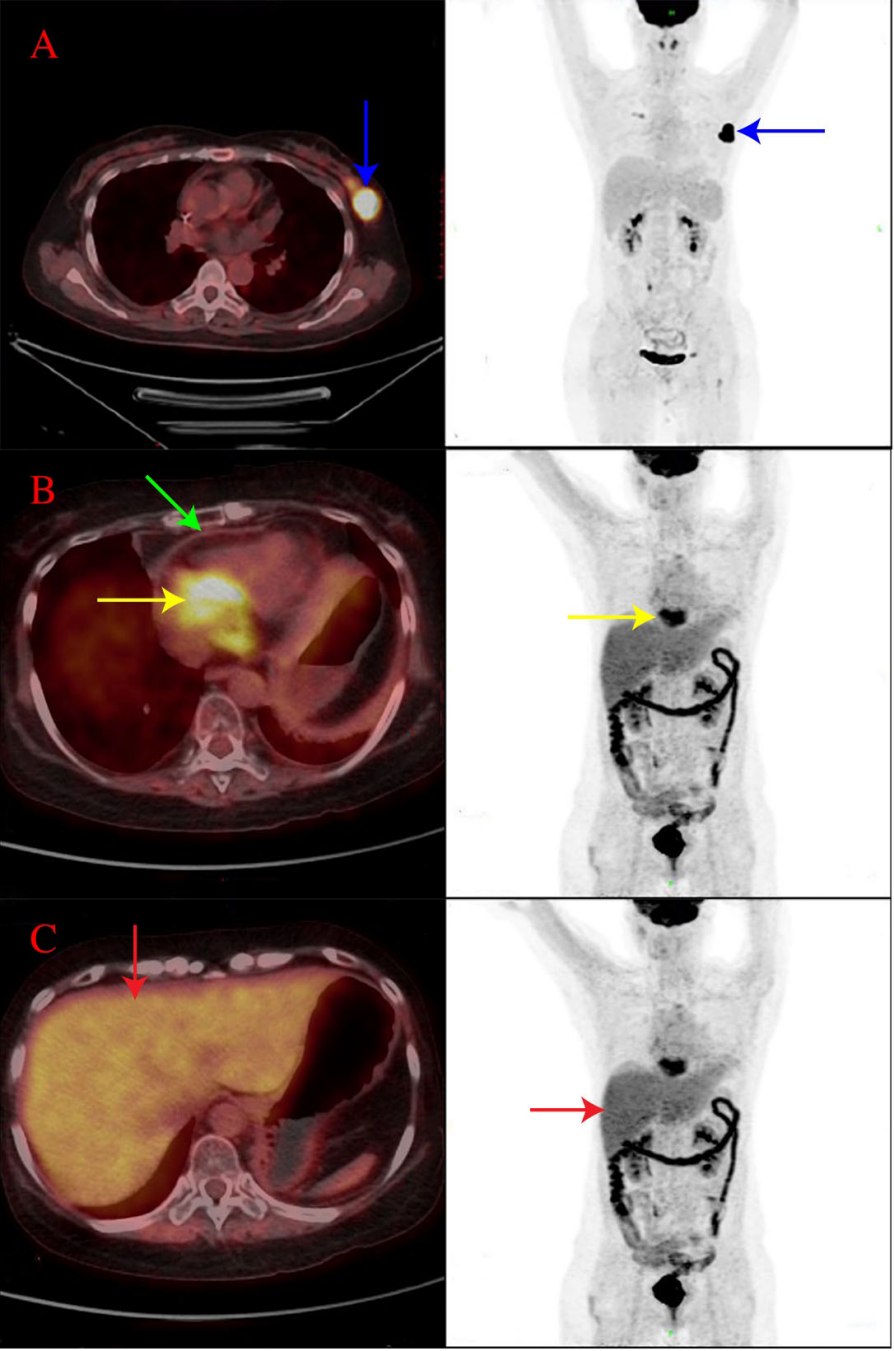
PET-CT findings of the patient. **(A)** In September 2020, there was an irregular soft tissue mass in the upper outer quadrant of the breast (4.1 × 2.0 cm), with an SUV_max_ of 23.0, as shown by the blue arrow. **(B)** In November 2021, there were a cardiac mass (6.0 × 4.4 cm), with an SUV_max_ of 10.5, as shown by the yellow arrow and a diffusely thickened pericardium, with an SUV_max_ of 2.0, as shown by the green arrow. **(C)** In November 2021, there was an abnormal uptake in the liver, with an SUV_max_ of 5.0 and a Deauville score of 5 points, as shown by the red arrow.

In September 2020, the patient complained of left breast pain that had begun 1 week earlier. Breast ultrasound showed multiple nodules in the left breast, while IHC of a left breast biopsy specimen indicated extramedullary recurrence of B-ALL after transplantation. Neither bone marrow aspirate nor flow cytometry showed any abnormal blasts, while E2A/PBX1 was undetectable. PET-CT revealed progressive disease, with a maximum standard uptake value (SUV_max_) of 23.0 and a Deauville score of 5 points (**Figure 1A**). Then, the administration of decitabine, fludarabine, cytarabine, and granulocyte colony-stimulating factor was initiated. Acute graft-versus-host-disease of the skin occurred after donor lymphocyte infusion. Eventually, the breast nodules decreased in size, and PET-CT revealed PR of the disease, with a Deauville score of 4 points.

In November 2020, the patient underwent donor-derived anti-CD19 CAR T-cell infusion after a conditioning regimen with fludarabine and cyclophosphamide. The sequence of CD19 CAR consists of an anti-CD19 single-chain antibody fragment (FMC63), CD28 transmembrane domain, 4-1BB, CD3zeta, T2A autocleavage sequences, and endodomain-deleted EGFR (tEGFR) (12). Four days later, the patient had a recurrent high fever with a temperature of 40°C, along with lethargy and vomiting. Based on a previous study, a grade-2 cytokine release syndrome (CRS) was identified (13). These clinical issues improved after providing supportive care with antibiotics (meropenem and caspofungin) and dexamethasone. Meanwhile, no clear etiological evidence with next-generation sequencing (NGS) or blood culture was obtained. The patient achieved CR and was negative for minimal residual disease on the 14th day after CAR T-cell therapy and remained well for 5 months.

In May 2021, the patient complained of chest pain that worsened when the patient breathed in or lay flat. Electrocardiogram (ECG) and myocardial enzyme levels were normal. Echocardiography (Echo) revealed massive pericardial effusion with rapid progression to cardiac tamponade. Ultrasound-guided pericardiocentesis, which was performed to alleviate the clinical symptoms and drain the excess fluid around the heart, revealed the presence of a hemorrhagic and exudative liquid. Multiple diagnostic tests for pericardial effusion, including bacterial gram staining, bacterial culture, fungal culture, acid fast bacteria staining, tuberculosis (TB) antibody examination, biopsy, and NGS, were performed. The results of all the above mentioned tests were negative. Flow cytometry of pericardial effusion and bone marrow aspirate did not reveal any tumor cells or CAR T cells. However, quantitative real-time polymerase chain reaction (qPCR) revealed that the copy number of CAR DNA per microgram of genome was 1.92 × 10^1^. The results of additional laboratory tests, including those of cytokines, are shown in **Table 1**. Given that some cytokines of the pericardial fluid were notably increased, CRS was considered as the possible cause of pericardial effusion. The pericardial effusion was gradually reduced and removed after immunosuppressive therapy with methylprednisolone and rucotinib. Bone marrow aspirate and PET-CT showed CR, with a Deauville score of 1 point.

**Table 1:** Cytokines in pericardial effusion and peripheral blood.

Cytokines (pg/ml)	IL-6	IL-8	IL-10	IL-17	IL-12P70	TNF-α	IFN-γ
Pericardial effusion	35,742.86	1,514.66	122.41	0.36	3.71	7.76	8.74
Peripheral blood	8.22	12.9	5.03	37.35	8.62	12.68	7.55

IL, interleukin; TNF-α, tumor necrosis factor-α; IFN-γ, interferon-γ.

On presentation to our hospital (November 2021), the patient complained of dyspnea and fatigue that had begun 2 weeks earlier. The patient’s medical history included hypertension. However, the patient did not need to take medications on a regular basis to control it under the guidance of a cardiologist. Immunosuppressive therapy with rucotinib and dexamethasone and preventive fungal treatment with orally administered posaconazole were continued. There were no known drug allergies. The patient lived with her husband and one child in rural China. The patient had previously worked as a farmer and did not smoke or drink.

**Figure 2:**
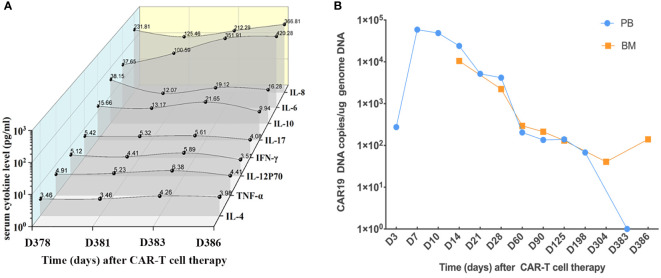
Serum cytokines and CAR19 DNA. **(A)** Serum cytokines of the patient during CAR T-cell therapy since admission. **(B)** CAR19 DNA in PB and BM since CAR T-cell therapy.

On examination, the patient’s temperature was 36.6°C, her heart rate was 104 beats per minute, blood pressure was 94/68 mmHg, respiratory rate was 25 breaths per minute, and oxygen saturation was 93% while the patient breathed ambient air. Severe edema of the limbs was identified, while the results of the remaining examinations were normal. The copy number of cytomegalovirus (CMV) was 8,953 per milliliter (reference range: <1,000). Other laboratory test results are shown in **Table 2**. ECG revealed changes in the ST segment, and Echo revealed that the left ventricular ejection fraction (LVEF) had decreased to 49%. The level of serum cytokines was significantly increased (**Figure 2A**). Chest CT revealed interstitial inflammation of both lungs.

**Table 2:** Patient characteristics on admission.

Laboratory data^a^		
Variable	On presentation	Reference rangeThis hospital^b^
White cell count (per μl)	1,640	4,000–10,000
Differential count (per μl)		
Neutrophils	1,430	2,000–7,000
Lymphocytes	170	800–4,000
Monocytes	40	120–1,000
Eosinophils	0	20–500
Basophils	0	0–100
Hemoglobin (g/L)	77	110–150
Platelet count (per μl)	22,000	100,000–300,000
Hematocrit (%)	23.7	37–48
Total protein (g/dl)	5.23	6.6–8.3
Albumin (g/dl)	3.14	3.5–5.2
Globulin (g/dl)	2.09	2.0–3.5
Aspartate aminotransferase (U/L)	48.70	0–35
Alanine aminotransferase (U/L)	60.30	0–35
Alkaline phosphatase (U/L)	176.30	30–120
Glutamyl transpeptidase (U/L)	291.80	8–57
Lactate dehydrogenase (U/L)	1,150.20	0–248
Total bilirubin (mg/dl)	0.9	0–1
Creatinine (mg/dl)	0.75	0.55–1.0
Cardiac troponin I (ng/ml)	0.059	<0.04
Creatine kinase isoenzyme-MB (ng/ml)	7.5	0.5–5.0
B-type natriuretic peptide (pg/ml)	352	0–100

a To convert the values for bilirubin to micromoles per liter, multiply by 17.1. To convert the values for creatinine to micromoles per liter, multiply by 88.4.

b Reference values are affected by many variables, including the patient population and the laboratory methods used. They may therefore not be appropriate for all patients.

The patient received diuretic, albumin infusion, antiviral with ganciclovir, and supportive treatments. As part of CAR T-cell therapy, tocilizumab was administered to control elevated serum cytokine levels. Clinical symptoms were slightly improved within 1 week of admission. Notably, the level of cardiac troponin I (cTNI) was elevated prior to the administration of tocilizumab.

On the 12th day after presentation, PET-CT of the body and head was performed, which showed cardiac involvement and abnormal uptake in the liver (**Figures 1B, C**). Bone marrow aspirate and cytogenetic analysis were normal, while flow cytometry revealed lymphoblasts that accounted for 0.15% of the marrow cellularity. E2A/PBX1 rearrangement was also positive. The results of CAR T cells are shown in **Figure 2B**. A cardiac mass biopsy was needed to achieve the pathological diagnosis but was not performed due to the patient’s poor physical condition.

**Figure 3:**
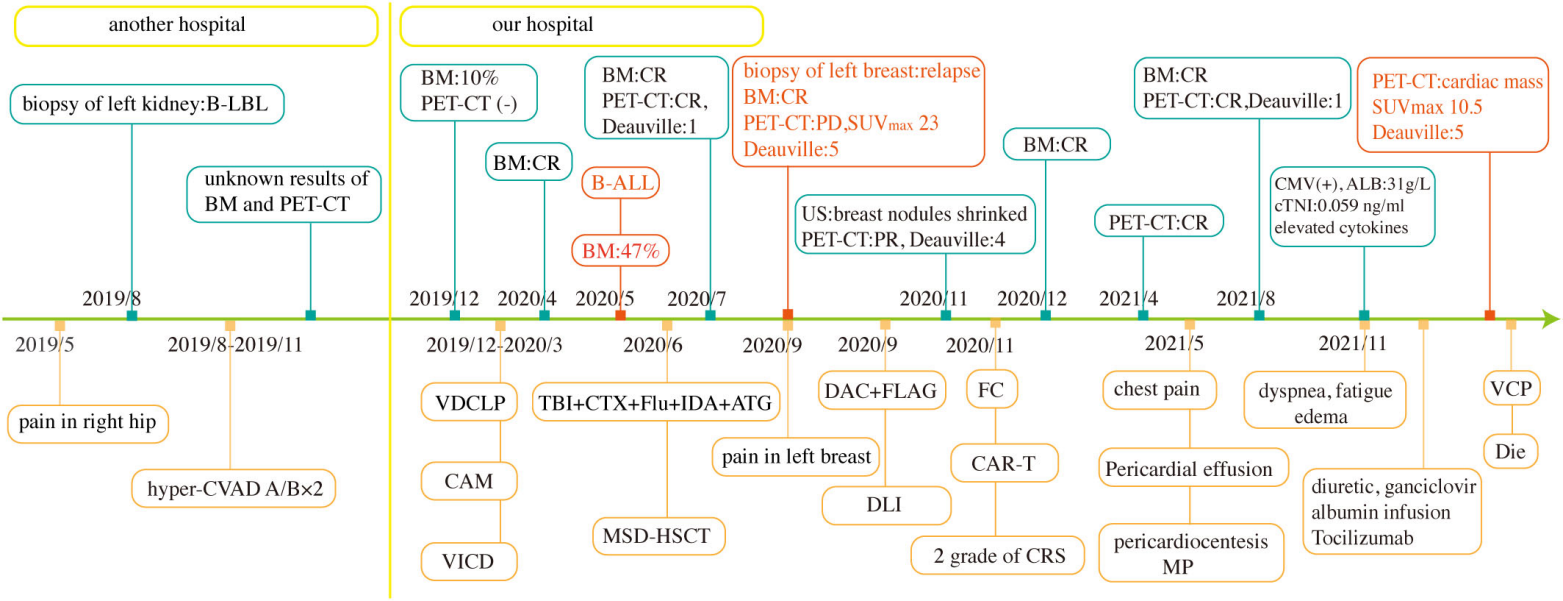
Clinical course of the patient. TBI+CTX+Flu+IDA+ATG, total body irradiation, cyclophosphamide, fludarabine, idarubicin, and antithymocyte globulin. BM, bone marrow aspirate; US, ultrasound; MP, methylprednisolone.

Owing to her poor physical condition, the patient was finally administered a reduced intensity chemotherapy with cyclophosphamide, vindesine, and prednisolone (VCP). Nevertheless, on the third day of chemotherapy, her blood pressure and oxygen saturation suddenly dropped to 61/29 mmHg and 76%, respectively. Along with a heart rate of 170 beats per minute, moist rales could be heard at the base of the left lung. ECG revealed tachycardia, ST-segment depression, and inverted T waves in the V2–6 leads. Although timely first-aid measures were provided, the patient could not be rescued and was eventually discharged from the hospital. The clinical course of the patient is shown in **Figure 3**.

## Discussion

Cardiac involvement is rare in patients with B-LBL/B-ALL, with only a few cases having been reported in the literature. It thus remains unclear how to identify cardiac involvement quickly and provide effective therapies. Some summative studies have demonstrated that cardiac metastases are detected in many cases at the time of postmortem (4, 5). Given the rarity of performing postmortems, only a few cases of cardiac involvement have been reported. We reviewed recent clinical cases with cardiac involvement and identified some common clinical manifestations (14–16). First, patients with cardiac involvement mostly presented non-specific symptoms, such as chest pain, dyspnea, and edema. Second, non-invasive imaging techniques, such as ECG, Echo, enhanced CT, cardiac magnetic resonance (CMR), and PET-CT, generally produced abnormal images, and PET-CT appears to have a diagnostic value in differentiating benign and malignant cardiac tumors and predicting prognosis, especially for cardiac metastases and lymphoma (17–19). Infiltration of leukemia cells into the myocardium may lead to cardiac conduction system abnormalities and rhythmic disturbances (20, 21), which mainly included non-specific ST–T wave changes and atrial arrhythmias. Although CMR and PET-CT are not always available, detection of cardiac involvement has benefited from the development of imaging technology in recent years. Unfortunately, the Echo and CT of this patient did not provide adequate imaging evidence of relapse.

For most metastatic cardiac tumors, surgery is not suitable, whereas surgery is a treatment option in case of primary cardiac tumors. Radiation therapy and chemotherapy have been routinely used to treat patients with cardiac metastases (3, 22). In a report by Kakefuda et al., a 61-year-old woman with B-ALL who received hyper-CVAD chemotherapy due to a cardiac mass finally achieved clinical remission (16). In addition, Manabe et al. reported that a 17-year-old patient with B-LBL with an intracardiac mass and myocardial infiltration underwent autologous peripheral blood stem cell transplantation after intensive chemotherapy and finally achieved long-term remission for over 5 years (15). Kahwash et al. reported that a 51-year-old patient with B-ALL with an infiltrative cardiac mass treated with palliative chest radiation ultimately died (14). The case presented in the current study had clinical symptoms similar to those of the above cases but a completely different course of disease progression. Thus, the treatment strategy for this patient differed slightly from those reported previously; this patient was administered tocilizumab and chemotherapy.

In the present study, we report a case of massive pericardial effusion close to tamponade, which occurred 6 months after CAR T treatment, and did not find common infectious causes of pericardial effusion, such as viruses, bacteria, fungi, and parasites, and non-infectious causes, including systemic inflammatory diseases, metabolic diseases, and drug-related factors, were also not considered (23). Although we found CAR DNA copies and abnormal increases in cytokines in pericardial effusion, no tumor cells or masses were found using flow cytometry or CT. The reason behind the occurrence of pericardial effusion remained unknown at the time. However, on reviewing the disease course of the patient, we speculated that the patient may have already had cardiac involvement during pericardial effusion. The main reasons why malignant cells were not detected in the pericardial effusion sample were the small number of leukemia cells and the powerful ability of CAR T cells to target and kill leukemia cells. In addition, the bloody pericardial effusion also supported our speculation of tumor invasion because inflammatory effusions are mostly green or yellow-green. CAR DNA data showed the persistence of CD19 CAR T cells in the patient, and CAR T cell consistently played a role of immunologic surveillance. Nevertheless, when leukemia cells invade the heart again, CAR T cells cannot exert a strong antileukemia effect as before, and the process of infiltration is accelerated to eventually form a cardiac mass owing to the decreased ability of CAR T-cell expansion.

CRS and immune effector cell-associated neurotoxicity syndrome (ICANS) are frequently reported due to the unique and life-threatening toxicity associated with CAR T-cell therapy. The mechanisms of CRS and ICANS have been explored in detail (24, 25). However, cardiovascular (CV) events caused by CAR T-cell therapy have not been explored much, but CV should also be taken seriously with limited data (26, 27). Patients treated with CAR T-cell therapy experienced arrhythmias, decompensated heart failure, and CV-related death. Seventeen patients had a CV event about 21 days after CAR T-cell therapy, six patients died from CV-related causes, six had decompensated heart failure, five had a new arrhythmia, and some patients showed decreased LVEF after CAR T treatment. A high grade of CRS (grade ≥2) can increase the incidence of CV events, and an elevated troponin level has been reported as a common clinical event associated with CRS in patients receiving CAR T-cell therapy, which has an evident relationship with CV events (26). Our patient showed abnormal elevations in cTNI and myocardial enzyme levels on the first day of hospitalization, which needed to be differentiated from myocarditis and acute coronary syndrome. However, we surprisingly found that cTNI began to decrease with the administration of tocilizumab, so we believed that the initial elevation of cTNI in this patient was mostly due to CRS associated with CV events rather than leukemia infiltration-induced myocardial injury. Further studies on the mechanism of TNI and CRS are needed.

## Conclusions

To the best of our knowledge, this is the first report of cardiac involvement as an extramedullary manifestation of relapse in a patient with B-LBL/B-ALL treated with allo-HSCT and CAR T-cell therapy. Notably, pericardial effusion may be the first presentation of the underlying relapse of malignancy; hence, diagnostic testing should not be stopped after determining the cytology of pericardial effusion in patients exhibiting CRS after CAR T-cell therapy. The possibility of cardiac involvement needs to be considered in time. Furthermore, simultaneous elevations in serum cytokines and TNI should be considered to distinguish CV associated with CRS from cardiac involvement. There is a need to accumulate more number of clinical cases of cardiac involvement to rapidly identify authentic causes of atypical clinical symptoms. We hope that our experience deepens the understanding of clinicians regarding cardiac involvement in patients with B-BLB/B-ALL who have undergone allo-HSCT and CAR T-cell therapy.

## Data availability statement

The original contributions presented in the study are included in the article/supplementary material. Further inquiries can be directed to the corresponding authors.

## Ethics statement

Written informed consent was obtained from the individual(s) for the publication of any potentially identifiable images or data included in this article.

## Author contributions

YL, YC, and XC were involved in the care of the patient. YL obtained the clinical data and wrote the manuscript. YC, RZ, WZ, QM, AP, DY, YH, and JW reviewed the data. EJ, MH, and SF supervised the entire study. All authors contributed to the article and approved the submitted version.
